# Compositional processing in the recognition of Chinese compounds: Behavioural and computational studies

**DOI:** 10.3758/s13423-025-02668-8

**Published:** 2025-03-06

**Authors:** Cheng-Yu Hsieh, Marco Marelli, Kathleen Rastle

**Affiliations:** 1 Department of Psychology, Royal Holloway, University of London, Egham, UK; 2 Department of Psychology, University of Milano-Bicocca, Milan, Italy; 3 NeuroMI, Milan Center for Neuroscience, Milan, Italy

**Keywords:** Chinese word recognition, Compound processing, Compositional distributional semantics, Meaning construction

## Abstract

Recent research has shown that the compositional meaning of a compound is routinely constructed by combining meanings of constituents. However, this body of research has focused primarily on Germanic languages. It remains unclear whether this same computational process is also observed in Chinese, a writing system characterised by less systematicity of the meanings and functions of constituents across compounds. We quantified the ease of integrating the meanings of Chinese constituent characters into a compositional compound meaning using a computational model based on distributional semantics. We then showed that this metric predicted sensibility judgements on novel compounds (Study 1), lexical decision latencies for rejecting novel compounds (Study 2), and lexical decision latencies for recognising existing compounds (Study 3). These results suggest that a compositional process is involved in Chinese compound processing, even in tasks that do not explicitly require meaning combination. Our results also suggest that a generic statistical learning framework is able to capture the meaningful functions of Chinese compound constituents. We conclude by discussing the advantages of routine meaning construction during compound processing in Chinese reading.

**Supplementary information:**

The online version contains supplementary material available at 10.3758/s13423-025-02668-8.

Characters are the basic unit of meaning in the Chinese writing system. However, they do not typically occur in isolation; instead, characters tend to be compounded to convey a specific meaning (Arcodia, 2007; Myers, 2022). For example, the compound word for “rooster” 公雞 (/gōng jī/) is composed of characters associated with “male” (公 /gōng/) and “chicken” (雞 /jī/). This structure is especially evident when we consider the formation of new Chinese words. Rather than coining a new character and assigning a meaning to it, the Chinese writing system typically creates a novel combination of existing characters (Ceccagno & Basciano, 2007; Lei et al., 2021). For example, the recently-coined word 書評 (/shū píng/, “book review”) consists of 書 (/shū/, “book”) and 評 (/píng/, “comment”; Ceccagno, 2016). The prevalence of compounding in Chinese means that understanding how readers compute the intended meaning of familiar and unfamiliar compounds is an important question for psycholinguistic research.

The examples provided in the previous paragraph may make it seem as if the meaning of a compound is the sum of its constituents’ meanings. However, meaning computation in Chinese is not as simple as adding up the constituent meanings, because the position of each constituent often suggests its semantic role. In Germanic languages, around 86.5% of compounds, such as “teacup” in English (or “*Teetasse*” in German), comprise constituents acting as a modifier and a head (Krott et al., 2004; Scalise et al., 2009). The head (“cup”) denotes the compound’s syntactic and semantic category, while the modifier (“tea”) specifies a property of the head: a “teacup” is a “cup” used for “tea”. The importance of positional information becomes even more apparent in reversible compounds such as “houseware” versus “warehouse”. The prevalence of the modifier-head structure in Germanic languages allows us to understand the distinction between them: the former is a manufactured article while the latter is a building.

Unlike Germanic languages, Chinese exhibits a variety of word structures beyond the modifier-head structure (Ceccagno & Basciano, 2007; Ji & Gagné, 2007; Scalise et al., 2009). The modifier-head structure is still the most prevalent (about 47.8%; Ceccagno & Basciano, 2007) and it is often critical in interpreting reversible compounds such as 蜜蜂 (/mì fēng/, “honeybee”, in which the first character “honey” modifies the second “bee”) and 蜂蜜 (/fēng mì/, “honey”, in which the first character “bee” modifies the second “honey”). However, there are multiple other structures including left-headed, two-headed, and headless structures. For example, a headless compound 大小 (/dà xiǎo/, literal: “big and small”) meaning “size” does not involve 大 “big” modifying 小 “small”. The variety of word structures in Chinese means that it is often difficult to predict the meaning of a compound from its constituents (see, e.g., Ji & Gagné, 2007; Marelli, 2023). Moreover, the mapping between the meanings of characters and whole compounds is not consistently systematic, as Chinese characters frequently bear multiple meanings (Hsieh et al., 2024; Liu et al., 2007; Tsang & Chen, 2010). For instance, the character 公 /gōng/ can mean not only “male” (as in 公雞 /gōng jī/, “rooster”) but also “public” (as in 公寓 /gōng yù/, literal: “public accommodation”, or “blocks of flats”).

Low systematicity of constituent meanings, along with high variability of word structures, poses important questions about whether and how Chinese readers integrate the meanings of constituents in the processing of compounds. Recent research on Germanic languages has revealed that the ease of combining constituents to derive a compound meaning influences not only the comprehension but also the visual recognition (lexical decision) of familiar and unfamiliar compounds (Günther et al., 2020; Günther & Marelli, 2019, 2020). These results suggest that readers attempt to combine the meanings of constituents during compound processing, irrespective of whether this is *required* by the task. However, the lower degree of systematicity across Chinese compounds may mean that this form of compositional processing is less effective and therefore predicts the processing of compounds to a lesser degree. This study therefore sought to investigate (a) the extent to which the ease of integrating the meanings of Chinese constituent characters into a compositional compound meaning predicts human reading behaviour across different tasks, and (b) whether the same computational principles used to describe compound processing in Germanic languages can also be applied successfully in Chinese.

## Semantic composition underpinning compound processing

Research shows that multimorphemic words are analysed in terms of their constituents in visual word recognition and comprehension (e.g., Amenta & Crepaldi, 2012; Stevens & Plaut, 2022). There is substantial evidence that inflected (e.g., Rastle et al., 2015) and derived (e.g., Longtin et al., 2003; Rastle et al., 2004) words are decomposed rapidly in visual word recognition. Similarly, there is evidence that the processing of compound words involves the analysis of constituent-level information (Crepaldi et al., 2013; Günther & Marelli, 2016; Smolka & Libben, 2017). Early theories of compound processing focused on the notion that constituents become activated during compound processing, perhaps in the service of recognising and understanding the whole compound (e.g., Libben, 1998; Peng et al., 1999; Taft, 2003; Zwitserlood, 1994). However, there has been growing interest in the notion that compound processing involves a process whereby the compositional meaning of the compound is constructed by combining constituent meanings. Research suggests that this form of meaning construction may be attempted routinely even when the compositional meaning deviates substantially from the conventional, lexicalised meaning (e.g., “ladybird” is not literally a female bird; Fiorentino & Poeppel, 2007; Ji et al., 2011; Spalding & Gagné, 2014).

One line of research that supports this theory has demonstrated that a compound is harder to process if there is more discrepancy between the compositional meaning and the conventional one. For instance, Ji et al. (2011) found that it typically takes longer to process opaque compounds (e.g., “hogwash”) than transparent compounds (e.g., “eggshell”), especially in tasks where semantic composition is encouraged. The notion that compound processing involves semantic composition is also supported by the relational priming effect: sensibility judgements on target compounds (“snowball”, ball MADE OF snow) are faster when the target is primed by a compound sharing the same relation (“snowman”, man MADE OF snow), than when it is primed by a compound sharing a different relation (“snowboard”, board FOR snow; e.g., Gagné et al., 2009; Gagné & Spalding, 2009).

### Compositional distributional semantic models

Recent advances in distributional semantic models (DSMs) provide a means to study the computational underpinnings of semantic composition (Amenta et al., 2020). DSMs represent word meanings as high-dimensional vectors learned via analysis of the way that words co-occur in large text corpora (Jones et al., 2015; Landauer & Dumais, 1997; Mandera et al., 2017). Building upon these traditional DSMs, compositional DSMs such as the CAOSS model (Composition as Abstract Operation in Semantic Space; Marelli et al., 2017; see also Guevara, 2010) allow researchers to compute the meanings of multimorphemic words by combining learned high-dimensional representations of their constituents. Thus far, the CAOSS model has been applied to affixed words (“blacken”; Bonandrini et al., 2023), compound words (“blackmail”; Günther & Marelli, 2019, 2020), and adjective-noun phrases (“black car”; Guevara, 2010). The CAOSS model learns high-dimensional representations for the constituents of compounds by experiencing those constituents in existing compounds. This training process allows the representations of compound constituents to be associated with information about the way in which the position of those constituents shapes their meaning (e.g., that a constituent in the first position is likely to modify the meaning of the constituent in the second position). In cognitive terms, this model asserts that readers develop position-specific knowledge about compound constituents through experience of whole compounds. Readers can then make sense of familiar and unfamiliar compounds through a process of combining those learned constituents.

The CAOSS model contrasts with a simple additive model (Mitchell & Lapata, 2010), which directly combines semantic representations of standalone words (i.e., learned outside of compound contexts) to derive a compound meaning without considering positional information. Unlike the CAOSS model, this simpler approach has difficulty capturing the roles of constituents when constituents appear in different positions, yielding the exact same representations for reversible compounds. The distinction between these two compositional DSMs is theoretically important in elucidating the computational nature of semantic composition. Specifically, the CAOSS model effectively captures the subtle variations in how the position in which a character occurs influences its meaning and function, whereas this is not the case for the simple additive model.

One important feature of compositional DSMs is their capability of representing the compositional meanings for both familiar and unfamiliar compounds. These model-derived representations enable us to quantify the ease with which compound meanings can be derived from the meanings of their constituents. Empirical studies have found that the *proximity* of the constituent representations to the compound representation (i.e., a measure describing how close in meaning a constituent is to the meaning of the whole compound) predicts lexical decision latencies for compounds (Günther & Marelli, 2019) and novel compounds (Günther et al., 2020; Günther & Marelli, 2020). These findings suggest that the ease with which the meaning of a compound can be derived from its constituents predicts reading behaviour even in tasks that do not require semantic analysis, speaking to the automaticity of this process. These findings also suggest that the meaningful functions of compound constituents are sufficiently consistent in the compounds in which they are encountered as to be learned by the CAOSS model (Günther & Marelli, 2023; Marelli et al., 2017).

### Research aims

Previous studies have shown that constituents are readily accessed during recognition of Chinese compounds (e.g., Hsieh et al., 2024; Tsang & Chen, 2013; Xiong et al., 2023). However, it remains unclear whether and how readers consider the meanings of individual characters *in combination* during the processing of compounds. The high degree of ambiguity in the meanings of Chinese characters (Hsieh et al., 2024) and the much greater degree of structural variability in Chinese compounds (Ceccagno & Basciano, 2007) raise questions about whether this type of compositional process would foster efficient understanding in Chinese. Likewise, while the CAOSS model provides an account of the influence of semantic composition in the recognition of English and German compounds (Günther et al., 2020; Günther & Marelli, 2019, 2020), it is unclear whether there is sufficiently consistent structure in Chinese compounding for the generic CAOSS architecture to learn the meaningful functions of Chinese compound constituents.

This study therefore aimed to investigate the extent to which the ease of combining constituents in deriving a compound meaning influences the comprehension and visual recognition of Chinese compounds. Specifically, we investigated whether there is evidence of semantic composition across different tasks that vary in the extent to which they require semantic composition: sensibility judgements on novel compounds (Study 1), lexical decision rejection latencies for novel compounds (Study 2), and lexical decision latencies for existing compounds (Study 3). We also sought to investigate whether the CAOSS model can be extended to Chinese reading, despite its lower degree of systematicity, and whether it provides a superior account of compound processing than a simple additive model (Mitchell & Lapata, 2010).

## Method

To investigate semantic composition in compound processing, we examined the performance of the CAOSS model (Marelli et al., 2017) against human behavioural data. This model involves (a) the transformation of free-standing characters into compound constituents based on their position-specific usage within compound words and (b) the combination of these transformed constituents to compute the meaning of a whole compound. In this study, we trained this model on existing two-character compounds derived from a corpus of traditional Chinese script, using a publicly available DSM for traditional Chinese script. In addition, we compared the CAOSS model with a baseline, the simple additive model (Mitchell & Lapata, 2010), which simulates the direct combination of character meanings into a compound, without the need for additional supervised learning processes designed to capture the position-specific functions of compound constituents.

From each model, we derived *proximity measures* characterising the relationship between constituent meanings and the induced compound meanings. We then sought to determine the relationship between these model-driven measures and various forms of human behavioural data to understand how the ease of incorporating character meanings into overall compound meaning impacts how adults process compounds (Günther & Marelli, 2019, 2020). We hypothesised that the proximity measures derived from the CAOSS model would perform better than those derived from the simple additive model due to the importance of positional information in Chinese (Hsieh et al., 2024). Below, we describe how we trained these computational models and how we derived measures from these models.

### Data availability

The data, analysis scripts and materials for this study are available at https://osf.io/8qzdx/. This study was not preregistered.

### Character and word vectors from the word2vec model

Our compositional DSMs were built using the word2vec model (Mikolov et al., 2013) pretrained on traditional Chinese developed by Academia Sinica, Taiwan (available at https://ckip.iis.sinica.edu.tw/project/embedding). We chose this model as it shows good performance in simulating human semantic relatedness judgements (Hsieh et al., 2024). This model was trained using the skip-gram with negative sampling (SGNS) method, which predicts word contexts given a target character or word, and 300-dimensional vectors for 500,000 characters and words can be obtained in this model. The character vectors derived from this model represent their individual, standalone forms.

### Training materials for compositional models

The current study focused on the semantic composition of constituent characters in two-character compound words, as this is the most common type of compound in Chinese (Shi, 2002; Zhou & Marslen-Wilson, 1995). To train compositional DSMs, we located 33,675 two-character compounds (which constitute approximately 91% of the total word tokens) in the corpus of traditional Chinese script developed by Hsieh et al. (2024). This corpus merges three previously published corpora: the Ministry of Education, Taiwan (National Language Committee, 2000), Academia Sinica, Taiwan (Chinese Knowledge Information Processing Group, 2001), and Wu and Liu (1988). However, we excluded compounds with whole-word frequency and character frequency lower than 10^1^ in the combined corpus due to their potentially poor representations in the word2vec model (Turian et al., 2010).

To substantiate this decision, we computed the semantic transparency of each compound word in the corpus (i.e., cosine similarity between the whole word vector and the character vector) and analysed the extent to which this was in line with human semantic transparency ratings (i.e., the extent to which the meaning of a compound is semantically related to its individual characters on a scale of 1 to 7) under different frequency exclusion thresholds. Human semantic transparency judgements were sourced from Tse et al. (2017). The data presented in Table 1 suggest that the correlation between model and human data is lowest when the frequency threshold is less restrictive, and that the correlation increases up to the frequency ≤10 threshold. These data suggest that adopting a frequency ≤10 threshold provides the optimal balance between model performance and the desire to retain as many observations as possible. In the end, 20,149 compounds were left for training the compositional DSMs (drop rate = 40.2%) once the frequency threshold was implemented.

**Table 1:** Spearman’s rank correlations (ρ) between the semantic transparency measures from human ratings (Tse et al., 2017) and that from the word2vec model given different exclusion criteria

Exclusion criterion	Number of observations (in proportion to the whole dataset)	Spearman’s ρ for C1	Spearman’s ρ for C2
Freq ≤1	21,995 (87.0%)	.416	.376
Freq ≤5	18,783 (74.3%)	.429	.387
Freq ≤10	16,188 (64.0%)	.434	.391
Freq ≤20	13,060 (51.7%)	.433	.393
Freq ≤50	8,519 (33.7%)	.432	.381

*Note*. The exclusion criterion is a threshold set to exclude items with a certain raw frequency count of compounds and constituents. C1 = first character; C2 = second character; Freq = raw frequency count in the combined corpus developed by Hsieh et al. (2024)

### Compositional models

In this study, we built two different compositional DSMs using the DISSECT toolbox (Dinu et al., 2013). One is the CAOSS model, which represents compounds by assigning different weights to the two word2vec character vectors before combining them. The compositional compound vector of the CAOSS model (*c*_*CAOSS*_) is formally expressed as:

1 $${c}_{CAOSS}={W}_{CI }{\nu }_{CI}+{W}_{C2 }{\nu }_{C2},$$

where *v*_*C1*_ and *v*_*C2*_ are the word2vec vectors of the first and second character as standalone units, respectively, and *W*_*C1*_ and* W*_*C2*_ denote the position-specific weight matrices for the first and second character, respectively. For example, the CAOSS-derived compound vector for 公雞 (/gōng jī/, “rooster”) is the sum of the vector of 公 (/gōng/, “male” or “public”) multiplied by *W*_*C1*_ and the vector of 雞 (/jī/, “chicken”) multiplied by* W*_*C2*_. The weight matrices transform free-standing characters into position-specific constituents whose meanings can be combined to yield compositional representations of compound words (including novel compound words). Estimation of these two matrices is the primary objective of the CAOSS model.

The other model is the simple additive model (Mitchell & Lapata, 2010), serving as a baseline model in this study. Its compositional compound vector (*c*_*additive*_) is expressed as:

2 $${c}_{additive}={\nu }_{CI}+{\nu }_{C2},$$

where *v*_*C1*_ and *v*_*C2*_ are the word2vec vectors of the first and second character as standalone units, respectively. This model captures how readers make sense of compound meanings by simply aggregating the meanings of individual standalone constituents. However, this approach may have difficulty in capturing the nuanced differences between constituents embedded in different positions as mentioned in Introduction.

The CAOSS model was trained on 20,149 existing two-character compounds along with their individual constituent characters. These compounds were not annotated with their types of internal structure during the training of the model, which simulates the learning of compound representations where prior meta-linguistic knowledge of different compound types is not typically involved.^2^ During the training, two 300-by-300 matrices, *W*_*C1*_and* W*_*C2*_, were estimated by minimising the discrepancy between the existing semantic vectors of the compounds induced by the word2vec model and their compositional counterparts obtained by the CAOSS model (*c*_*CAOSS*_), following Formula 1. These matrices were then applied to create compositional vectors for both existing and novel compounds.

### Probing semantic composition: proximity measures

We computed *proximity measures* that represent the closeness between individual character meanings and the model-derived compound meaning. These measures are used to examine the ease with which constituent meanings combine to derive overall compound meaning in compound processing (Günther & Marelli, 2019, 2020). If it is easy to engage in a composition process, that means readers tend to use the meanings of characters to make sense of compound meaning. The two measures used in this study are formally defined below and each measure can be computed for the first and the second character of a two-character Chinese compound:

1. CosSim(*c*_CAOSS_, *W*_*C1*_* v*_*C1*_) and CosSim(*c*_CAOSS_, *W*_*C2*_* v*_*C2*_): the cosine similarity between the CAOSS-derived compositional compound vector (*c*_CAOSS_) and the word2vec vectors of its individual constituents (*v*_*C1*_, *v*_*C2*_) multiplied by the position-specific matrices (*W*_*C1*_, *W*_*C2*_).
2. CosSim(*c*_additive_, *v*_*C1*_) and CosSim(*c*_additive_, *v*_*C2*_): the cosine similarity between the additive-derived compositional compound vector (*c*_additive_) and the word2vec vectors of its individual constituents (*v*_*C1*_, *v*_*C2*_).

The first measure, under the framework of the CAOSS model, involves the process where readers combine character meanings with their positional information considered. By contrast, the second measure, under the framework of the simple additive model, captures the process where readers directly combine meanings of the two characters into a compound without considering their positions and/or functions. Comparing these two measures helps us determine how readers use character meanings in deriving compositional compound meanings. The distinction also enhances our understanding of whether it is necessary to transform a Chinese character from free-standing units (*v*_*C1*_, *v*_*C2*_), to constituents (*W*_*C1*_* v*_*C1*_, *W*_*C2*_* v*_*C2*_) embedded in a compound (*c*_*CAOSS*_) before combining character meanings.

### Analysis scheme using behavioural measures

To probe the semantic composition process, we examined the influence of these proximity measures against three behavioural measures from psycholinguistic tasks: sensibility judgements on novel compounds (Study 1), lexical decision latencies for rejecting novel compounds (Study 2), and lexical decision latencies for recognising existing compounds (Study 3). In each analysis, we first determined which semantic proximity measures—those from the CAOSS model or those from the simple additive model—provided the best fit to the data using Akaike information criterion (AIC). A better model fit is indicated by smaller values of AIC. Linear mixed-effect models using the selected proximity measures were fitted on the behavioural measures probed in each analysis using the *lme4* package in R using maximum likelihood estimation (Bates et al., 2015). Potential transformation of the raw data was suggested using Box–Cox transformation analysis (Box & Cox, 1964), and the normality of regression residuals was examined using Q–Q plot. We do not report these analyses, but they are available in R code on the OSF site for this project. In addition to the proximity measures of C1 and C2, we also included (log-transformed) family size of C1 and C2 in the model, since this factor is known to influence compound processing (e.g., Hsieh et al., 2024; Kuperman et al., 2009; M.-F. Li et al., 2015). We defined family size as the number of Chinese compounds that share the same character at a specific position. This metric was extracted from the corpus developed by Hsieh et al. (2024).

In each linear mixed-effect model, main effects of (log-transformed) C1 family size, C1 proximity, (log-transformed) C2 family size and C2 proximity were centred to the mean before being entered as predictors (Iacobucci et al., 2016). Additionally, we introduced random intercepts for C1 and C2. For the analysis involving word data (i.e., Study 3), we also included (log-transformed) whole-word frequency as a control variable. Similar to the training of computational models, we excluded all items whose word frequency or constituent frequency was lower than 10. We then reported the best-performing model, along with *p* values obtained using the Satterthwaite approximation in the *lmerTest* package in R (Kuznetsova et al., 2017) and the semipartial *R*^2^ obtained using the *r2glmm* package in R (Jaeger, 2017).

## Study 1: Sensibility judgements on novel compounds

In this study, we investigated the extent to which a compositional process influences participants’ judgements about the meaningfulness of novel two-character compounds.

### Participants

Participants were 30 native speakers of Taiwanese Mandarin, a language using the traditional Chinese writing system. They were recruited through social media platforms, specifically targeting individuals from the UK and Taiwan. The participants comprised 11 men and 19 women, with ages ranging from 25 to 40 years (*M* = 28.3 years, *SD* = 3.1). None had a previous history of language or reading disorder. Compensation of £5 or 200 New Taiwan Dollars (about £5) was offered for their participation in the study. This study was approved by the University Research Ethics Committee at Royal Holloway, University of London.

### Material and design

We randomly selected 1,500 novel compounds from Tse et al. (2017), a dataset that includes approximately 25,000 novel compounds created by randomly combining two existing characters in traditional Chinese script. This selection of items had similar distributions of lexical properties, including C1 family size, C1 proximity, C2 family size, and C2 proximity, as the entire dataset (see more details in Supplementary Material B on the OSF site for this project). These items were further divided into three subsets, each of which consists of 500 novel compounds. Participants were randomly assigned to one of the subsets. Participants were requested to make a sensibility judgement on each item based on how easy it was to come up with a potential meaning, using a 5-point Likert-type scale, with 1 being *almost impossible* and 5 being *extremely easy*. Participants were also instructed that there was no correct response for each item. The testing was conducted via Google Forms.

To ensure participants’ agreement on sensibility ratings, an intraclass correlation (ICC) (McGraw & Wong, 1996) was computed for each subset of items using the *irr* package in R (Gamer et al., 2019). The resulting ICCs ranging from 0.55 to 0.76 indicated moderate-to-good consistency (Cicchetti, 1994; Koo & Li, 2016), suggesting that participants provided a reasonable degree of agreement on sensibility judgements across items. Average sensibility ratings were calculated for each novel compound and, following the Box–Cox transformation analysis and inspection of the Q–Q plot, were entered into the model without any transformation.

### Results

The CAOSS-derived proximity measures produced a better fit to the model and were therefore selected as predictors (see Table 2). The subsequent linear mixed-effects analysis revealed that all the main effects were significant (*p* values < .0001; see Table 3): C1 family size, C2 family size, C1 proximity, and C2 proximity. The direction of these effects is such that higher family size and higher proximity increase the perceived sensibility of the novel compounds.

**Table 2:** AIC values of different proximity measures in each study

Study	CosSim(*c*_additive,_ *v*)	CosSim(*c*_CAOSS_, *Wv*)
1 (*n* = 1,478)	2645	**2640**
2 (*n* = 22,553)	−40871	**−40889**
3 (*n* = 15,145)	−19215	**−19265**

*Note.* Models with better fit (i.e., lower AIC values) are in bold. CosSim = cosine similarity; *c* = compound word vector derived from the simple additive model or the CAOSS model; *v* = word2vec character vector; *W* = CAOSS-derived weight matrix

**Table 3:** Effects of family size and proximity measures on sensibility ratings for novel compounds (Study 1), rejection latencies for novel compounds (Study 2), and response latencies for existing compounds (Study 3)

Study	Dependent variable	Predictor	Estimate	*SE*	*t*	*p*	Semipartial *R*^2^ (%)
1	Sensibility ratings for novel compounds	Intercept	2.533	0.017	150.04	<.0001	–
		Log-C1 FS	0.141	0.019	7.63	<.0001	4.2
		C1 proximity	0.956	0.212	4.51	<.0001	1.5
		Log-C2 FS	0.082	0.015	5.59	<.0001	2.2
		C2 proximity	0.792	0.271	2.92	.0035	0.6
2	Inv-RT of novel compounds	Intercept	1.382	0.002	783.49	<.0001	–
		Log-C1 FS	−0.026	0.001	−20.15	<.0001	4.7
		C1 proximity	−0.196	0.014	−13.75	<.0001	1.7
		Log-C2 FS	−0.016	0.001	−15.24	<.0001	2.4
		C2 proximity	−0.090	0.017	−5.29	<.0001	0.2
3	Inv-RT of existing compounds	Intercept	1.585	0.002	791.26	<.0001	–
		Log-word freq	0.045	0.001	55.94	<.0001	16.8
		Log-C1 FS	0.014	0.002	8.23	<.0001	0.7
		C1 proximity	0.131	0.016	8.38	<.0001	0.7
		Log-C2 FS	0.016	0.002	10.56	<.0001	1.5
		C2 proximity	0.199	0.020	9.71	<.0001	1.1

*Note.* Inv-RT = inversely-transformed reaction times; C1 = first character; C2 = second character; log = log-transformed; word freq = whole word frequency; FS = family size

## Study 2: Lexical decision latencies for novel compounds

The task in Study 1 required participants to derive a meaning for the novel compound. Studies 2 and 3 investigate whether a compositional process arises in a task in which it is not required: deciding whether a two-character compound is an existing word or not. Study 2 focuses on the extent to which a compositional process influences response times for rejecting novel compounds in a lexical decision task.

### Material and design

We used rejection latencies for novel compounds from an existing megastudy of lexical decision (Tse et al., 2017), in which 33 Cantonese speakers in Hong Kong responded to each item. Novel compounds whose accuracy across participants was less than 70% were removed (Tse et al., 2017; Tse & Yap, 2018), leaving 23,807 novel compounds (drop rate = 5.1%). Further, we removed items whose proximity measures could not be calculated due to no available semantic vectors for their constituent characters, and whose constituent frequency fell below the threshold (i.e., raw frequency less than 10), leaving 22,553 novel compounds (drop rate = 3.8%) in the analysis. Response times were inversely transformed before being entered into the model (based on the Box–Cox analysis and inspection of the Q–Q plot).

### Results

The CAOSS-derived proximity measures produced a better fit to the model and were therefore selected as predictors (see Table 2). The subsequent linear mixed-effects analysis revealed that all the main effects were significant (*p *values < .0001; see Table 3): C1 family size, C2 family size, C1 proximity, and C2 proximity. The direction of these effects was such that higher family size and higher proximity values slowed rejection latencies for novel compounds.

We focused on response time data because the accuracy rates were high (*M* = 90%) and were highly negatively skewed (*Mdn* = 94%). However, we still analysed the accuracy of novel compounds using the same model design in order to ensure that there was no evidence of a speed-accuracy trade-off. The analysis of accuracy mirrored the analysis of response time (higher family size and higher proximity values reduced accuracy), thus excluding the possibility of a speed-accuracy trade-off. The analysis of accuracy is reported in Supplementary Material C on the OSF site for this project.

## Study 3: Lexical decision latencies for existing compounds

In the previous two analyses, novel compounds were used as stimuli. In this section, we instead used existing compounds to investigate the influence of a semantic composition process in the presence of lexical representation.

### Material and design

This analysis was conducted on lexical decision latencies of approximately 25,000 two-character compound words from Tse et al. (2017). Compounds whose accuracy across participants was less than 70% were removed (Tse & Yap, 2018; Tse et al., 2017), leaving 22,808 words (drop rate = 9.8%). We further excluded items whose proximity values could not be calculated due to no available semantic vectors for their constituent characters, or whose constituent frequency and whole-word frequency fell below the frequency threshold (i.e., raw frequency less than 10), leaving 15,145 compounds (drop rate = 32.2%) in the analysis. Response times were inversely transformed prior to being entered into the model (based on the Box-Cox analysis and inspection of the Q–Q plot).

### Results

Table 2 indicates that the CAOSS-derived proximity measures outperformed the proximity measures derived from the additive model.^3^ We therefore entered the CAOSS-derived proximity measures into the linear mixed-effects analysis reported in Table 3. The analysis revealed that all the main effects were significant (*p *values < .0001): C1 family size, C2 family size, C1 proximity, and C2 proximity. The direction of these effects was such that the recognition of compounds was faster with higher family size and higher proximity.

We focused on response time data because accuracy rates were high (*M* = 92%) and were highly negatively skewed (*Mdn* = 97%). However, we still analysed the word accuracy data using the same model design to ensure that there was no evidence of a speed-accuracy trade-off. These analyses showed a similar pattern as the response time data, with higher family size and higher proximity increasing accuracy, thus excluding the possibility of a speed-accuracy trade-off. The analysis of accuracy is reported in Supplementary Material C on the OSF site for this project.

### Discussion

The processing of compound words has long been an important object of study in the psychology of language (Libben, 1998; Peng et al., 1999; Taft, 2003; Zwitserlood, 1994). Recent research has focused on the notion that lexical processing may involve the routine combination of meaningful constituents. These inferences derive from the observation that compound representations induced from the CAOSS model (a compositional DSM) influence behaviour in lexical processing tasks (Günther et al., 2020; Günther & Marelli, 2019, 2020). However, these findings and the application of compositional DSMs have been confined to Germanic languages. This study therefore sought to investigate the extent to which semantic composition influences compound processing in Chinese, a writing system with far less systematicity in the meaningful functions of compound constituents.

Our primary finding was that the ease of integrating constituent meanings into a compositional meaning impacts comprehension of novel compounds (Study 1), the rejection of novel compounds in lexical decision (Study 2), and the recognition of existing compounds in lexical decision (Study 3). As meanings of individual constituents contribute more to the compositional meaning of compounds (i.e., higher proximity), readers require less cognitive effort in their attempt to combine constituent meanings (Günther & Marelli, 2019, 2020). Previous research has shown that morphological effects can be sensitive to task demands (Duñabeitia et al., 2011; Marelli et al., 2013), but our results take this further in showing that a semantic composition process arises in Chinese compound processing, and even in tasks in which it is not required. These findings are in line with the theory of “maximisation of opportunity” in which readers endeavour to activate all available cues to access a compound meaning (Libben, 2014), even though conflicts between the compositional meaning and the conventional (lexical) meaning may slow processing (Fiorentino & Poeppel, 2007; Ji et al., 2011; Spalding & Gagné, 2014). This approach makes sense when we consider Chinese reading, as readers are continuously encountering compounds they have not seen before in situations where the goal is understanding (Li & Zhou, 2012).

Our findings also reveal that the CAOSS model provides a good description of compositional processing in Chinese, and that it outperforms an additive model in which compound representations are derived by adding standalone vectors for the constituent characters together.^4^ Despite the varied word structures in Chinese, the CAOSS model effectively simulates a process of statistical learning, acquiring information about how position shapes the meanings of compound constituents through exposure to compound words and their meanings in the environment. The statistical regularities learned can then be applied to the combination of lexicalised and novel compounds. The successful implementation of the CAOSS model in Chinese is broadly in line with a body of research suggesting that rules of combining meaningful units are acquired through experience with multiword chunks (see Christiansen & Arnon, 2017, for review).

Thus far, it seems that Chinese lexical processing patterns with findings from Germanic languages. However, there is a subtle difference between Germanic languages and Chinese regarding the effect of the ease of integrating constituents into a compound. In Germanic languages, the effects observed are mainly associated with the modifier (i.e., the first constituent), rather than the head, suggesting a more important role of modifier in guiding compound processing (Günther et al., 2020; Günther & Marelli, 2019, 2020; Marelli et al., 2017; Spalding et al., 2010). However, our findings reveal significant proximity effects for both characters, suggesting both characters are important during compound processing. These findings are consistent with previous research: for example, Ji and Gagné (2007) found relational priming effects from both constituents on Chinese compound processing, while (Cui et al., 2021) found equivalent processing times for each character in Chinese compounds using eye tracking. The apparent cross-linguistic difference here may be attributed to the difference in the variability of word structures (Ji & Gagné, 2007). Because Chinese compounds do not have a consistent structure, Chinese readers may have to determine the word structure after considering both constituents.

Another potential reason for semantic composition in Chinese is that the meaningfulness of constituents *in combination* may help to determine word boundaries in sentences. There are no spaces between words in Chinese, and as such, the problem of how readers determine lexical boundaries is a long-standing issue (He et al., 2021; Huang & Li, 2024). One potential strategy is to derive meaningfulness of multiword chunks through semantic composition. Consider a three-character Chinese compound, 打籃球 (/dǎ lán qiú/, “play basketball”). It is best parsed into 打 (“hit” or “play”) and 籃球 (“basketball”), rather than 打籃 (“hit basket”) and 球 (“ball”). The decision for parsing might be due to an underlying compositional process that indicates combining 打 (“hit” or “play”) and 籃 (“basket”) is less meaningful than combining 籃 (“basket”) and 球 (“ball”). This issue requires further exploration from a compositional and semantic perspective, as it could provide insights into the word segmentation challenge during Chinese sentence comprehension.

In conclusion, the present study suggests that the processing of both familiar and unfamiliar Chinese compounds routinely involves a semantic composition process. Our computational work further suggests that readers can learn the meaningful functions of constituent characters through exposure to whole compounds, and that the computational principles underpinning this learning process are similar to those in Germanic languages, despite the much lower systematicity of Chinese compounding.

## Supplementary information

Below is the link to the electronic supplementary material.

Supplementary file1 (PDF 554 KB)

## Data Availability

The data and materials for this study are available at: https://osf.io/8qzdx/

## Footnotes

1 The robustness of this decision was examined through a series of additional analyses. More details can be found in Supplementary Material A available on the OSF site for this project.

2 Previous research has shown that even without morpho-syntactic information during training, the semantic features captured by the CAOSS model still distinguish different compound word structures (Pezzelle & Marelli, 2020).

3 One reviewer wondered how CAOSS-derived proximity measures performed against human ratings of semantic transparency. We found that (a) CAOSS-derived proximity measures provided a better fit to lexical decision latencies than human ratings, and (b) CAOSS-derived proximity measures increased the variance explained by our regression model when human ratings were included. Further details can be found in Supplementary Material D on the OSF site for this project.

4 One reviewer of this article queried whether this result held up when interactions between proximity and family size were included in the statistical models. We report this exploratory analysis in Supplementary Material E on the OSF site for this project and show that our conclusions remain broadly unchanged.

## Code availability

The analysis scripts for this study are available at: https://osf.io/8qzdx/
