# Supplementary material for: Compositional processing in the recognition of Chinese compounds: Behavioural and computational studies
Source: Psychon Bull Rev. 2025 Mar 6;32(4):1878–89. doi: 10.3758/s13423-025-02668-8 (PMC12325552; doi:10.3758/s13423-025-02668-8)
Supplement: Supplementary file 1 — Supplementary file1 (PDF 554 KB) [file 13423_2025_2668_MOESM1_ESM.pdf]

Supplementary Material for "Compositional processing in the recognition of Chinese compounds: Behavioural and computational studies" published at *Psychonomic Bulletin & Review*

Table of Contents

Supplementary Material A: Robustness of Studies 1 Through 3 2

Supplementary Material B: Lexical Properties of the Stimuli in Study 1 4

Supplementary Material C: Analyses on Accuracy Data in Lexical Decision 8

Supplementary Material D: Comparison with Human Ratings of Semantic Transparency 12

Supplementary Material E: On Adding Interactions to our Analyses.................................... 13

Supplementary Material A: Robustness of Studies 1 Through 3

In Studies 1 through 3, we only used compound words with word frequency and constituent frequency larger than 10 for training compositional models and for analysing the behavioural measures. To ensure the robustness of the model comparison analyses, we reran the study using different exclusion criteria for raw frequency of characters and words. The application of different frequency criteria applied both to the materials used for training the compositional models (see Column 2 in Table 1) and to the testing set used for the model comparison analysis (Table 2).

Results showed that the pattern of data reported in Table 2 is broadly replicated across different frequency exclusion thresholds (see Table A1): the CAOSS-derived measures are superior to the additive-derived measures. Exceptions arose when the threshold of frequency ≤1 and ≤5 was applied in Study 2; however, such a low frequency threshold risks poor representations in the vector space (Turian et al., 2010; see also Table 1). The additive-derived measures also performed better than the CAOSS-derived when the threshold of frequency ≤50 is applied in Study 3. However, the analyses required the exclusion of around 63% of the data, likely compromising the validity of the result.

Table A1

AIC values of proximity measures across different frequency thresholds for exclusion

Frequency Threshold	1	5	10	20	50
n of Analysis 1	1500	1492	1478	1459	1373
CosSim($c_{additive}$, *v*)	2682	2668	2645	2601	2454
CosSim(c_{CAOSS}, ***W****v*)	**2677**	**2665**	**2640**	**2595**	**2450**
n of Analysis 2	22946	22774	22553	22114	20849
CosSim($c_{additive}$, *v*)	**-41551**	**-41258**	-40871	-40129	-37929
CosSim(c_{CAOSS}, ***W****v*)	-41512	-41231	**-40889**	**-40159**	**-37986**
n of Analysis 3	20314	17503	15145	12183	7934
CosSim($c_{additive}$, *v*)	-26175	-22434	-19215	-15333	**-9778**
CosSim(c_{CAOSS}, ***W****v*)	**-26189**	**-22486**	**-19265**	**-15368**	-9768

Note. Models with better fit (i.e. lower AIC values) are in bold. CosSim = cosine similarity; *c* = compound word vector derived from the additive model or the CAOSS model; *v* = character vector; ***W*** = CAOSS-enriched matrix.

Supplementary Material B: Lexical Properties of the Stimuli in Study 1

In Study 1, we have selected 1,500 novel compounds from an existing megastudy that contains around 25,000 novel compounds (Tse et al., 2017). We have checked that the selected items had similar distributions of lexical properties, including C1 family size, C1 proximity, C2 family size, and C2 proximity, as the entire dataset. In this file, we presented the density plots of these lexical properties (Figures B1-B4).

Figure B1

Distributions of (a) C1 Family Size (FS) and (b) C2 Family Size (FS) in the entire dataset of Tse et al. (2017)

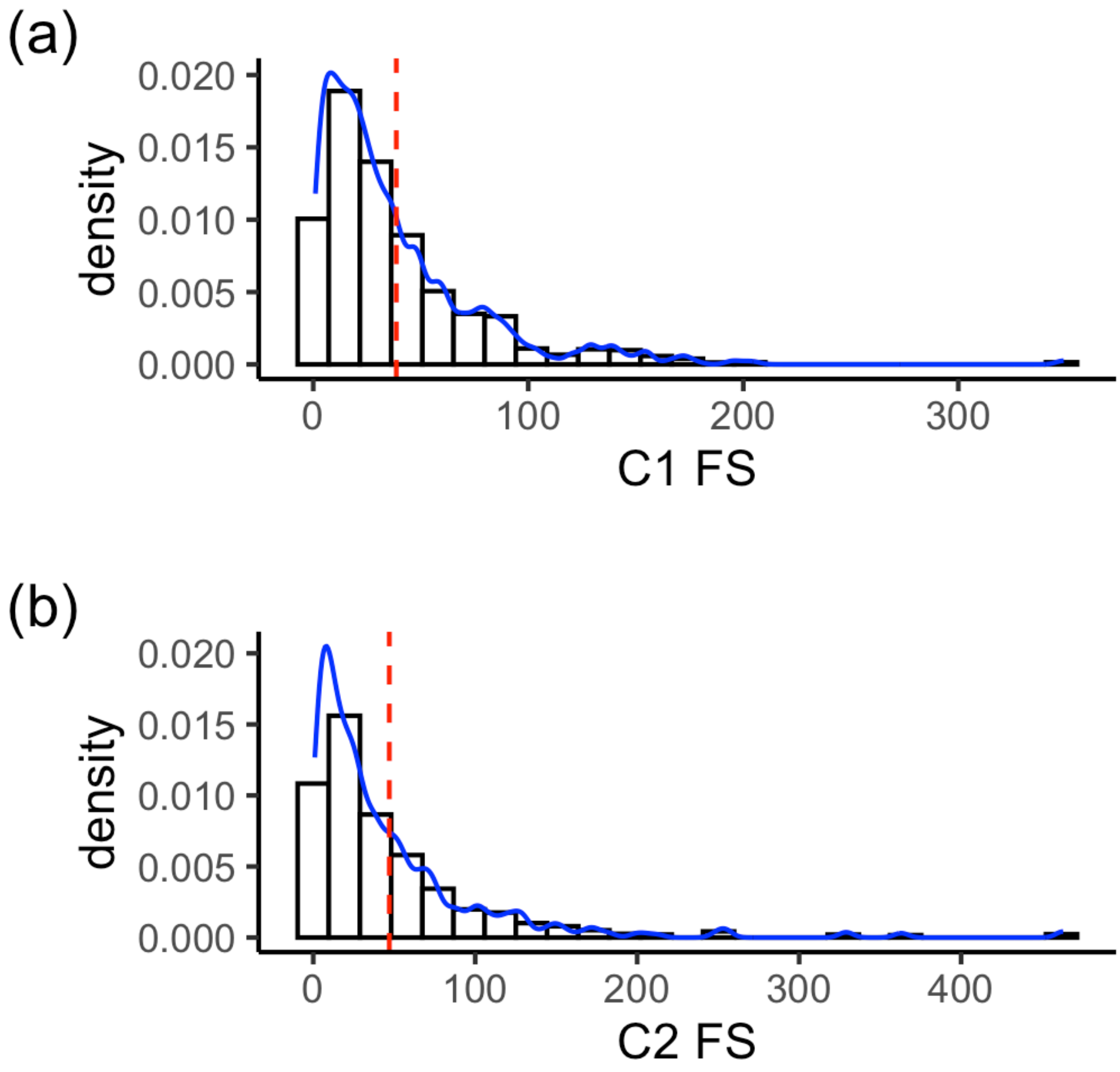

Figure B2

Distributions of (a) C1 Family Size (FS) and (b) C2 Family Size (FS) in the stimuli for Study 1

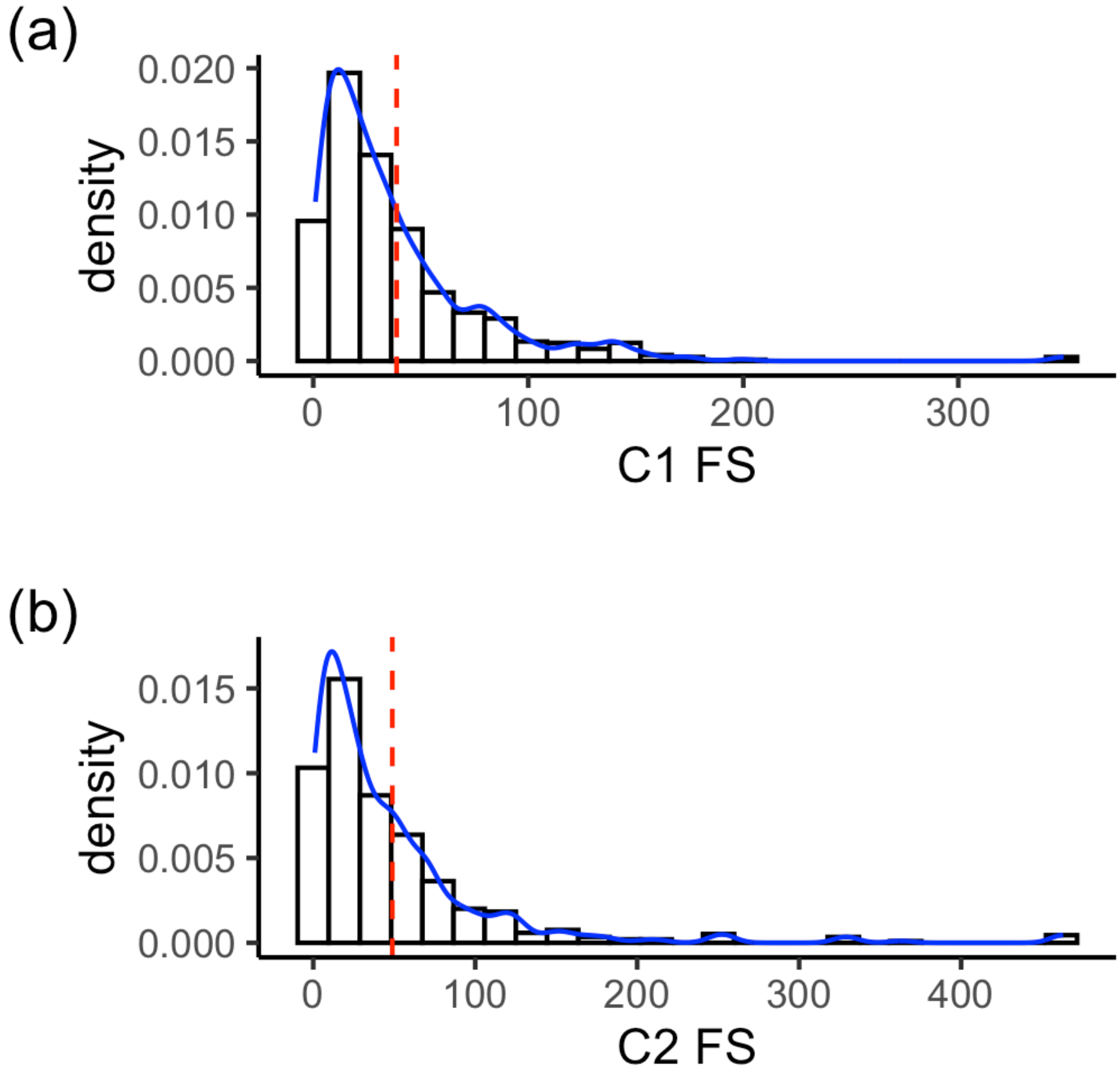

Figure B3

Distributions of (a) C1 Proximity and (b) C2 Proximity in the entire dataset of Tse et al. (2017)

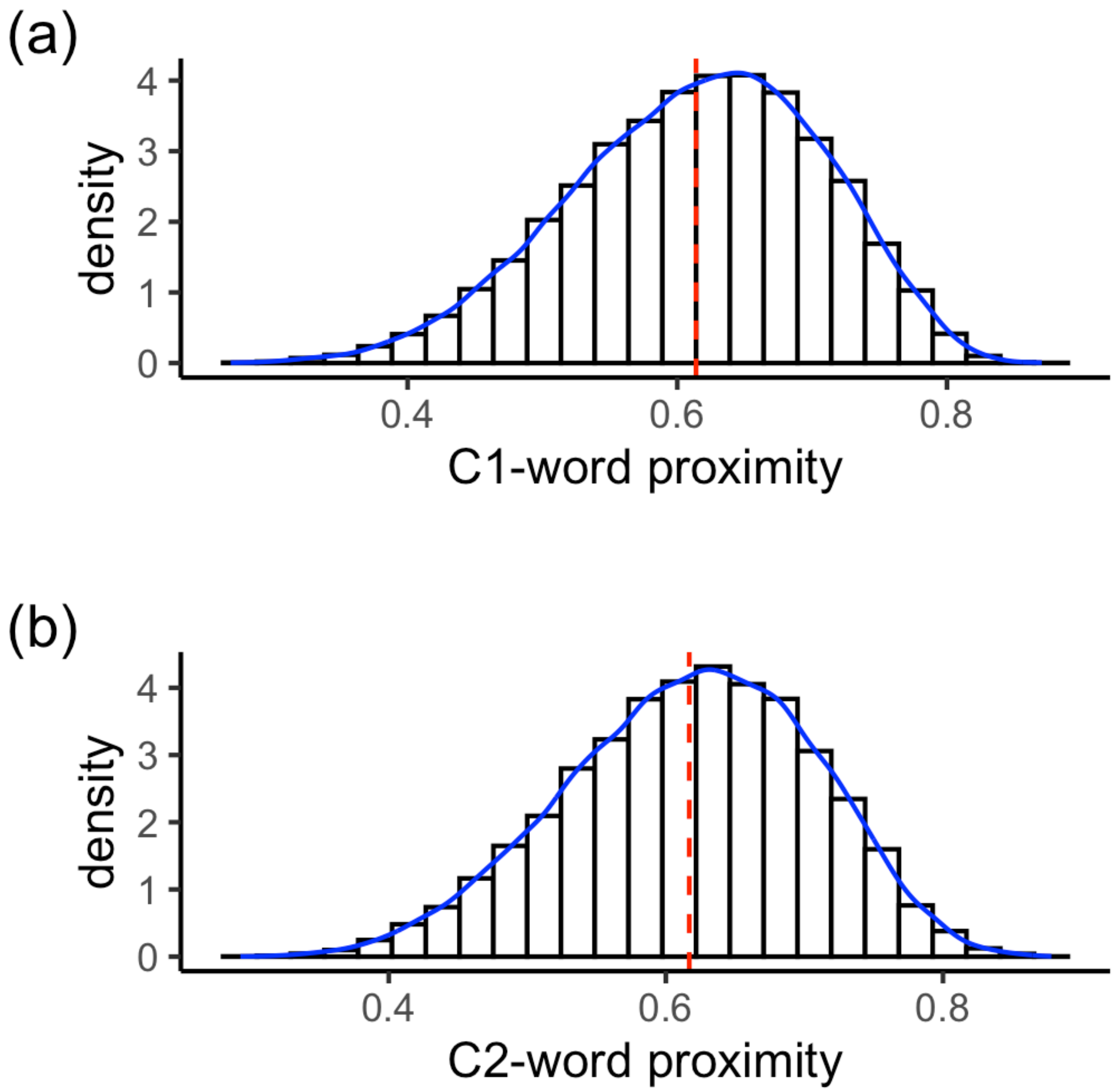

Figure B4

Distributions of (a) C1 Proximity and (b) C2 Proximity in the stimuli for Study 1

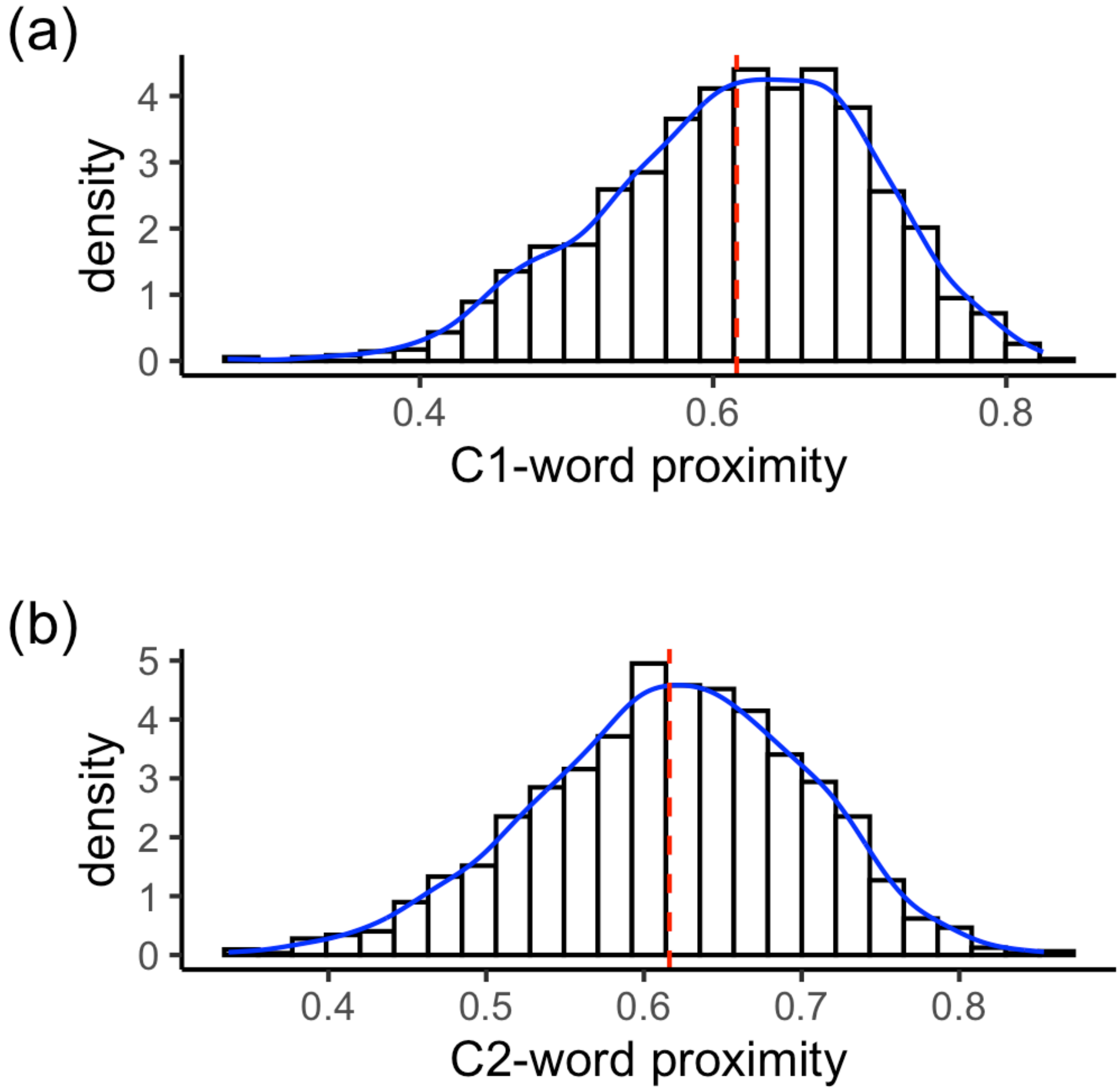

Supplementary Material C: Analyses on Accuracy Data in Lexical Decision

To check the presence of a speed-for-accuracy tradeoff, we also analysed the accuracy data of novel compounds and existing compounds using the same model design.

Novel Compound Accuracy Analysis

Methods

We used novel compound rejection accuracy (i.e. correct rejection rate) from an existing megastudy of lexical decision (Tse et al., 2017), in which 33 Cantonese speakers in Hong Kong responded to each item. We removed novel compounds whose proximity measures could not be calculated due to no available semantic vectors for their constituent characters, and whose constituent frequency fell below the threshold (i.e. raw frequency less than 10), leaving 23,728 novel compounds (drop rate = 5.41%) in the analysis. The average accuracy rate for these novel compounds is 90.0%, with standard deviation of 11.0%. The data was highly negatively skewed, with a median of 94%.

A generalised linear mixed-effect model was fitted on the number of correct and incorrect responses for novel compounds using the glmmTMB package in R (Brooks et al., 2017). Following Hsieh et al. (2024), a logit link function with a beta-binomial family was used for the model (Agresti, 2012; Harrison, 2015) considering the overdispersion present in the data. In line with the model design for latency analysis (Study 2), the main effects of C1 proximity, (log- transformed) C1 family size, C2 proximity, and (log- transformed) C2 family size centred to the mean (Iacobucci et al., 2016) and entered as predictors in the model. Note that the proximity measures were both derived from the CAOSS model according to the model comparison analysis (see Table 2).

Results

Table C1 shows that all the main effects were significant (*p*s < .0001): C1 family size, C2 family size, C1 proximity and C2 proximity. The direction of these effects was such that higher family size and higher proximity values make rejecting novel compounds less accurate. This aligned in the direction for reaction time, showing no evidence of a speed-accuracy tradeoff.

Existing Compound Accuracy Analysis

Methods

We used existing compound accuracy (i.e. hit rate) from an existing megastudy of lexical decision (Tse et al., 2017). We removed words whose proximity measures could not be calculated due to no available semantic vectors for their constituent characters, and whose constituent frequency fell below the threshold (i.e. raw frequency less than 10), leaving 15,937 words (drop rate = 37.0%) in the analysis. The average accuracy rate for these words is 92.1%, with standard deviation of 11.2%. The data was highly negatively skewed, with a median of 97%.

A generalised linear mixed-effect model was fitted on the number of correct and incorrect responses for words using the glmmTMB package in R (Brooks et al., 2017). Following Hsieh et al. (2024), a logit link function with a beta-binomial family was used for the model (Agresti, 2012; Harrison, 2015) considering the overdispersion present in the data. In line with the model design for latency analysis (Study 3), the main effects of C1 proximity, (log- transformed) C1 family size, C2 proximity, and (log- transformed) C2 family size centred to the mean (Iacobucci et al., 2016) and entered as predictors in the model, with (log-transformed) whole word frequency controlled. Note that the proximity measures were both derived from the CAOSS model according to the model comparison analysis (see Table 2).

Results

Table C1 shows that all the main effects were significant (*p*s < .0001): C1 family size, C2 family size, C1 proximity and C2 proximity. The direction of these effects was such that higher family

size and higher proximity makes the recognition of words more accurate. This aligned in the direction for reaction time, showing no evidence of a speed-accuracy tradeoff.

Table C1

Effects of family size and proximity measures on accuracy of novel compounds (Study 2), and accuracy of existing compounds (Study 3)

Study	DV	Predictor	Estimate	*SE*	*Z*	*p*	OR
2	Accuracy of novel compounds	Intercept	2.254	0.014	163.20	<.0001	-
		Log-C1 FS	-0.281	0.011	-24.44	<.0001	0.76
		C1 proximity	-1.757	0.130	-13.49	<.0001	0.17
		Log-C2 FS	-0.228	0.009	-25.07	<.0001	0.80
		C2 proximity	-0.990	0.154	-6.42	<.0001	0.37
3	Accuracy of existing compounds	Intercept	2.515	0.016	159.77	<.0001	-
		Log-word freq	0.254	0.008	32.34	<.0001	1.29
		Log-C1 FS	0.117	0.013	8.71	<.0001	1.12
		C1 proximity	1.789	0.128	14.01	<.0001	5.98
		Log-C2 FS	0.060	0.012	5.08	.002	1.06
		C2 proximity	1.772	0.163	10.85	<.0001	5.88

Note. DV = dependent variable; C1 = first character; C2 = second character; log = log-transformed; word freq = whole word frequency; FS = family size; OR = odds ratio.

References:

Agresti, A. (2012). *Categorical data analysis* (3rd ed.). John Wiley and Sons.

Brooks, M. E., Kristensen, K., Van Benthem, K. J., Magnusson, A., Berg, C. W., Nielsen, A., Skaug, H. J., Machler, M., & Bolker, B. M. (2017). glmmTMB balances speed and flexibility among packages for zero-inflated generalized linear mixed modeling. *The R Journal*, *9*(2), 378–400. https://doi.org/10.3929/ethz-b-000240890

Harrison, X. A. (2015). A comparison of observation-level random effect and beta-binomial models for modelling overdispersion in binomial data in ecology & evolution. *PeerJ*, *3*, Article e1114. https://doi.org/10.7717/ peerj.1114

Hsieh, C.-Y., Marelli, M., & Rastle, K. (2024). Beyond quantity of experience: Exploring the role of semantic consistency in Chinese character knowledge. *Journal of Experimental Psychology: Learning, Memory, and Cognition, 50*(5), 819–832. https://doi.org/10.1037/xlm0001294

Iacobucci, D., Schneider, M. J., Popovich, D. L., & Bakamitsos, G. A. (2016). Mean centering helps alleviate “micro” but not “macro” multicollinearity. *Behavior Research Methods*, *48*(4), 1308–1317. https://doi.org/ 10.3758/s13428-015-0624-x

Tse, C.-S., Yap, M. J., Chan, Y.-L., Sze, W. P., Shaoul, C., & Lin, D. (2017). The Chinese lexicon project: A megastudy of lexical decision performance for 25,000+ traditional Chinese two-character compound words. *Behavior Research Methods*, *49*(4), 1503–1519. https://doi.org/10.3758/s13428- 016-0810-5

Supplementary Material D: Comparison with Human Ratings of Semantic Transparency

One reviewer wondered how CAOSS-derived proximity metrics compared to human ratings of semantic transparency in predicting the lexical decision latencies in Study 3. We conducted two analyses to address this question (details available in R script on OSF). First, we compared our model against a model where human ratings (extracted from Tse et al., 2017) were entered as predictors instead. We found that CAOSS-derived proximity measures (AIC = -19520) offered a better fit to lexical decision latencies than human ratings (AIC = -19511). Second, we tested whether CAOSS-derived proximity measures increase the variance explained given the inclusion of human ratings. AIC, BIC, and the likelihood ratio test all suggest that the CAOSS-derived measures capture the variance that cannot be explained by human ratings (Table S4). These results suggest that computational metrics can serve as a better predictor than human ratings.

Table S4

Model comparison between models with and without CAOSS-derived proximity measures when human ratings of semantic transparency (extracted from Tse et al., 2017) were included

	Without Proximity	With Proximity
AIC	-19511	**-19582**
BIC	-19442	**-19498**
Likelihood Ratio Test	$X^2(2) = 74.9$, $p < .0001$	

Supplementary Material E: On Adding Interactions to our Analyses

One of the reviewers queried whether our main pattern of results held when we included an interaction between family size and proximity in our statistical models. We did not include an interaction term in our main analyses for two reasons. The first reason was that we did not have any *a priori* hypotheses about the effect of an interaction on our dependent variables. It has been well rehearsed that modifying statistical models – adding interactions, covariates, or other parameters— in the absence of any hypotheses is bad practice (Sijtsma, 2016; Wagenmakers et al., 2012). Such practice increases the likelihood of false positives, and because these analyses are not based on any hypotheses, it can be difficult to interpret any effects that do arise.

The second reason was that adding an interaction term risks providing the additive model with position-specific information that it is not meant to have. Our family size measures are computed in a position-specific manner because previous research has shown these to outperform family size measures computed in a position-generic manner (Hsieh et al., 2024). In allowing these measures to interact with proximity measures from the additive model, we risk introducing position-specific information into those measures and wiping away the very distinction that we are trying to evaluate.

Despite these strong reservations, to be fully transparent we report the model comparison analyses for all three studies below (Tables E1-E3), with and without the interaction term. We report both AIC and BIC but suggest that BIC is more appropriate given that the analyses are exploratory (as BIC penalises the addition of additional terms much more; McCoach et al., 2022).

Table E1

AIC and BIC values of models with and without the interaction term in Study 1

**Model**	**AIC**	**BIC**
CAOSS with interaction	2638	2691
CAOSS without interaction	2640	2682
Additive with interaction	2631	2683
Additive without interaction	2645	2687

Table E2

AIC and BIC values of models with and without the interaction term in Study 2

**Model**	**AIC**	**BIC**
CAOSS with interaction	-40906	-40825
CAOSS without interaction	-40889	-40825
Additive with interaction	-40891	-40810
Additive without interaction	-40871	-40807

Table E3

AIC and BIC values of models with and without the interaction term in Study 3

Model	AIC	BIC
CAOSS with interaction	-19268	-19184
CAOSS without interaction	-19265	-19197
Additive with interaction	-19227	-19143
Additive without interaction	-19215	-19147

These analyses show that the main pattern of results does not change for the larger Studies 2 and 3. The CAOSS model still has the best fit to the data, and there is no clear pattern as to whether the interaction helps, hurts, or does nothing to the fit. However, the pattern of results for the smaller Study 1 becomes somewhat ambiguous. Here, the interaction term substantially improves the fit of the additive model while doing nothing or hurting the CAOSS model, making it difficult to discriminate between the two models. It is difficult to interpret the ambiguous result for Study 1. This was a relatively small study, so it is possible that the findings are just more sensitive to model (mis)specification. The fact that the analysis with the interaction was exploratory also gives us less confidence in the validity of the findings. Finally, it is noteworthy that the inclusion of the interaction disproportionally benefits the additive model across all studies, at least when AIC values are considered. This points to the possibility that the interaction is providing useful position-specific information to the additive model that it is not meant to have.

Taken together, these exploratory analyses suggest that the CAOSS model robustly outperforms the additive model in the larger Studies 2 and 3. The result is less clearly supportive of the CAOSS model in the smaller Study 1, but we also do not have clear empirical or theoretical support for the additive model in this analysis.

References:

Hsieh, C.-Y., Marelli, M., & Rastle, K. (2024). Beyond quantity of experience: Exploring the role of semantic consistency in Chinese character knowledge. *Journal of Experimental Psychology: Learning, Memory, and Cognition, 50*(5), 819–832. https://doi.org/10.1037/xlm0001294

McCoach, D. B., Newton, S. D., & Gambino, A. J. (2022). Multilevel model selection: Balancing model fit and adequacy. In M. S. Khine (Ed.), *Methodology for Multilevel Modeling in Educational Research: Concepts and Applications* (pp. 29-48). Singapore: Springer Singapore.

Sijtsma, K. (2016). Playing with data—or how to discourage questionable research practices and stimulate researchers to do things right. *Psychometrika, 81*, 1-15. https://doi.org/10.1007/s11336-015-9446-0

Wagenmakers, E. J., Wetzels, R., Borsboom, D., van der Maas, H. L., & Kievit, R. A. (2012). An agenda for purely confirmatory research. *Perspectives on Psychological Science*, *7*(6), 632-638. https://doi.org/10.1177/1745691612463078
